# Non-Invasive 3D Breast Tumor Localization: A Viable Alternative to Invasive Tumor Marking

**DOI:** 10.3390/cancers16142564

**Published:** 2024-07-17

**Authors:** Dragana Bjelica, Natasa Colakovic, Svetlana Opric, Darko Zdravkovic, Barbara Loboda, Simona Petricevic, Milan Gojgic, Ognjen Zecic, Zlatko Skuric, Katarina Zecic, Nebojsa Ivanovic

**Affiliations:** 1 Department of Radiology, University Hospital Medical Center “Bežanijska kosa”, Zorza Matea bb, 11070 Belgrade, Serbia; draganastevanovic82@gmail.com; 2 Department of Surgical Oncology, University Hospital Medical Center “Bežanijska kosa”, Zorza Matea bb, 11070 Belgrade, Serbiaivanovicnebojsadr@gmail.com (N.I.); 3 Faculty of Medicine, University of Belgrade, Dr. Subotica 8, 11000 Belgrade, Serbia; 4 Department of Pathology, University Hospital Medical Center “Bežanijska kosa”, Zorza Matea bb, 11070 Belgrade, Serbia; 5 Faculty of Dentistry Pancevo, Zarka Zrenjanina 179, 26000 Pancevo, Serbia; 6 Department of General Surgery, University Hospital Medical Center “Bežanijska kosa”, Zorza Matea bb, 11070 Belgrade, Serbia; 7 Clinic for Gynecology and Obstetrics, Clinical Center of Serbia, Visegradska 26, 11000 Belgrade, Serbia

**Keywords:** early breast cancer, neoadjuvant systemic therapy, pathological complete response, surgical excision, marking techniques, tumor positioning, ultrasound

## Abstract

**Simple Summary:**

Tumor marking before neoadjuvant systemic therapy (NAST) is an essential part of early breast cancer treatment, aiming to enable adequate surgical excision of the tumor bed in cases with complete clinical regression. Standard surgical techniques for targeted excision generally rely on the invasive insertion of various markers (radiopaque clips, radioactive seeds, silver wire, etc.) into or around the tumor. These techniques are burdened by technical complexity, the risk of complications (hematoma, infection, marker migration, etc.), and limited precision in the insertion and later identification of markers. Herein, we present our original technique of non-invasive three-dimensional tumor localization before and repeatedly during NAST and the preliminary results of its application. Multiple repetitions (monitoring the occasionally eccentric pattern of tumor regression) without the risk of complications suggest a possible advantage over invasive marking techniques. Application in surgical centers lacking trained staff and equipment for invasive tumor marking may impact the quality improvement of post-NAST breast cancer surgery in those centers.

**Abstract:**

**Background:** We present a detailed description and the preliminary results of our original technique for non-invasive three-dimensional tumor localization in the breast, which was created as an alternative to standard invasive tumor marking before neoadjuvant systemic therapy (NAST), aiming to enable adequate surgery after complete tumor regression. **Methods:** A detailed description of the technique is provided in the main text. The technique’s feasibility and precision were assessed in a single-arm, prospective study based on the histological parameters of the adequacy and rationality of the excision of completely regressed tumor beds. **Results:** Out of 94 recruited patients, 15 (16%) were deemed unsuitable, mainly due to the tumors’ inadequate ultrasound visibility. Among the 79 processed patients, 31 (39%) had complete clinical regression after NAST and were operated on using our technique. The histological parameters of surgical precision (signs of tumor regression: 24/31; microscopic cancer residues: 7/31) were verified in all excised specimens (100% precision). There were no positive margins in seven cases with microscopic residues, indicating our technique’s capacity to enable oncologically safe post-NAST surgery. **Conclusions:** The proposed technique is feasible and satisfactorily accurate in determining the location of regressed tumors, thus representing an alternative to invasive tumor marking, especially in surgical centers lacking trained staff and equipment for invasive marking. The technique’s limitations are mainly related to the inadequate ultrasound visibility of the tumor.

## 1. Introduction

Neoadjuvant systemic therapy (NAST) for breast cancer was introduced into clinical practice to surgically treat initially inoperable locally advanced cancers [1]. The indications for NAST have expanded to include enabling more sparing surgery for primary operable tumors [2], whereby in the latest indications, an incomplete response to NAST (lack of achievement of a complete pathological response (pCR)) necessitates additional adjuvant systemic therapy [3,4].

Complete clinical tumor regression (CCR) after NAST presents surgeons with the unique challenge of adequately excising the tumor bed after the significant regression or complete disappearance of the breast tumor tissue when the area of the pre-existing tumor cannot be identified radiologically or by palpation. Surgical excision of the tumor bed is essential for histologically verifying the degree of regression or achieving a pCR. A lack of orientation regarding the pre-existing tumor’s location often leads surgeons to opt for mastectomy [5,6], which contradicts one of the main goals of NAST: enabling breast-conserving surgery (BCS).

Standard diagnostic and surgical procedures that enable adequate BCS in the case of achieving CCR rely on the placement of diagnostically identifiable markers into or around the tumor before starting NAST, which serve as a guide for surgical excision during surgery.

With the increasing therapeutic efficacy of NAST and the higher percentage of achieved pCR, tumor-marking techniques before starting NAST have gradually gained importance to enable the precise excision of the tumor bed after CCR.

Several pre-NAST marking techniques have been described in the literature: tattoos on the skin overlying the tumor tissue [7,8], placing a radiopaque clip at the center of the tumor [6,9,10,11,12], tumor marking with radioactive seeds [13,14,15,16,17], ROLL [17,18], bracketing with silver wire around the tumor [19,20], inserting a radiopaque coil [21], hydrogel markers [22], and radiofrequency tags [23].

All the tumor-marking techniques described share the characteristic of one-time application: the tumor is marked before the start of NAST and never again during. This could limit the determination of the differences in tumor regression patterns during NAST [24,25] and occasional eccentric regressions where residual cancer tissue may occur on the periphery of the marked zone after significant regression.

The variety of techniques may indicate differences in the technical capabilities of different medical centers but also that no technique is ideal. Each has its drawbacks, such as lacking a third dimension (i.e., skin tattoos), challenges in ideal marker positioning (all techniques), additional requirements and inconveniences of working with radioactive materials (i.e., ROLL and radioactive seed), limitations in orientation regarding the extent of the excision based on a single punctiform marker, marker migration during NAST, or poor visibility of markers post-NAST [26,27]. Different patterns of tumor regression during NAST (concentric or non-concentric) limit the accuracy of excising the tumor bed, regardless of the marking method. Bracketing is more reliable than central marking, but it is technically more demanding and requires a wider excision than might be necessary. All techniques except tattooing implicate an invasive approach with potential complications (e.g., hematoma or infection). Preoperative wire-needle (WNL) marking of the marker is necessary in some cases. Invasive markings require specially trained staff, additional technical equipment, and financial outlays for equipment and markers.

A recent meta-analysis reviewing published studies on different tumor-marking techniques before NAST concluded “There is limited evidence that tumor marking before neoadjuvant chemotherapy improves the rate of unsatisfactory margins or survival outcomes in patients undergoing BCS after NAST” [28].

We created an original non-invasive three-dimensional tumor localization technique before and during NAST to optimize sparing breast cancer surgery in the case of CCR. Our technique’s concept initially had one goal: the ability for repeated application during NAST to dynamically monitor the therapeutic effects, the regression pattern, and the tumor’s eventual eccentric regression. The technique can be applied in medical centers lacking trained staff and equipment for invasive marking, which indicates the possibility of improving the quality of post-NAST surgery in those centers via its application. The above characteristics indicate our technique’s potential usefulness as an additional or alternative to standard invasive marking techniques.

This paper aims to present our original technique to the professional public, with a detailed description of the technique, based on which it can be applied without additional education, and to present the preliminary results of its application in our study as a test of its feasibility.

## 2. Materials and Methods

As part of a prospective, single-arm clinical study currently being conducted on the clinical significance of intensive and frequent monitoring of the therapeutic effects of NAST, the diagnostic and surgical team of the UHMC “Bezanijska Kosa” developed an original technique for the non-invasive three-dimensional positioning of tumors in the breast by ultrasound before initiating NAST, designed to optimize the surgical excision of the tumor bed after complete clinical regression. This technique differs from standard pre-NAST tumor-marking techniques in lacking any physical marker to guide post-NAST surgery. Instead, a record of the three-dimensional spatial position of the tumor in the breast before and during NAST is used as a guide. All measurements were performed on a Samsung RS 80 ultrasound, which is part of the standard equipment for breast diagnostics at our medical center.

Inclusion/exclusion criteria were as follows: Patients with a solitary breast tumor of up to 5 cm in size and an N0/N1 axillary status, in whom, based on the stage of the disease or biological characteristics of the tumor, NAST was multidisciplinarily indicated, were included in the study irrespective of the age or the tumor subtype. Patients with multicentric breast tumors and those with tumors larger than 5 cm and an N2 axillary status were not included.

Demographic and baseline disease characteristics of the patients were recorded before starting the therapy. Preoperative systemic therapy consisted of a standard regimen of 4 cycles (every 3 weeks) of cyclophosphamide plus doxorubicin (AC) and 12 cycles of taxanes weekly. HER 2-positive patients (immunohistochemical score 3+ or ERBB2 amplification by in situ hybridization) received a standard regimen of anti-HER2 treatment for 12 weeks (Trastuzumab for N0 and Trastuzumab + Pertuzumab for N1 patients), beginning at the first cycle of taxane therapy. The endocrine therapy for hormone receptor-positive tumors started after the operation.

After NAST, radiologically visible (via ultrasound) or palpable residual tumor tissue serves as an adequate marker of the tumor site and the extent of excision. Therefore, our target group included only patients in whom complete clinical tumor regression (non-visible and non-palpable residues) occurred under NAST. The tumor bed’s surgical excision in those patients was performed exclusively using our original technique, testing its precision and oncological reliability.

The test of the procedure’s oncological reliability (surgical excision adequacy) consisted of histological reports on the presence of histological indicators of a pre-existing tumor in the excised specimen, such as edema, necrosis, vascularized hyalinization, foamy macrophages, lymphocytes, hemosiderin-laden macrophages, or the absence of normal ductal and lobular structures. Histological margin status is assessed by the presence of histologically unchanged breast tissue in all directions around the target focus [29].

An intermediate test of the surgery’s functionality and esthetic effects was the ratio of the resected tissue specimen volume to the pre-NAST tumor volume. The ratio was obtained as the quotient of these two volumes. The volumes were calculated as the product of three orthogonal dimensions of the specimen and the pre-NAST tumor. Values of less than 1 (one) indicated that the volume of the excised specimen was smaller than the volume of the pre-NAST tumor; values greater than 1 indicated that the volume of the excised specimen was greater than the volume of the pre-NAST tumor.

A delayed (prolonged) test of the procedure’s oncological reliability will be the long-term outcome, i.e., the frequency of local recurrences.

## 3. Technique Description

The tumor’s three-dimensional position in the breast was determined and recorded in measurement units relative to known landmarks: (1) the projection of the tumor’s central point on the skin, (2) the distance of the tumor’s superficial margin from the skin, and (3) the distance of the tumor’s deepest margin from the pectoral fascia.

### 3.1. Diagnostic Procedure

**Figure 1:**
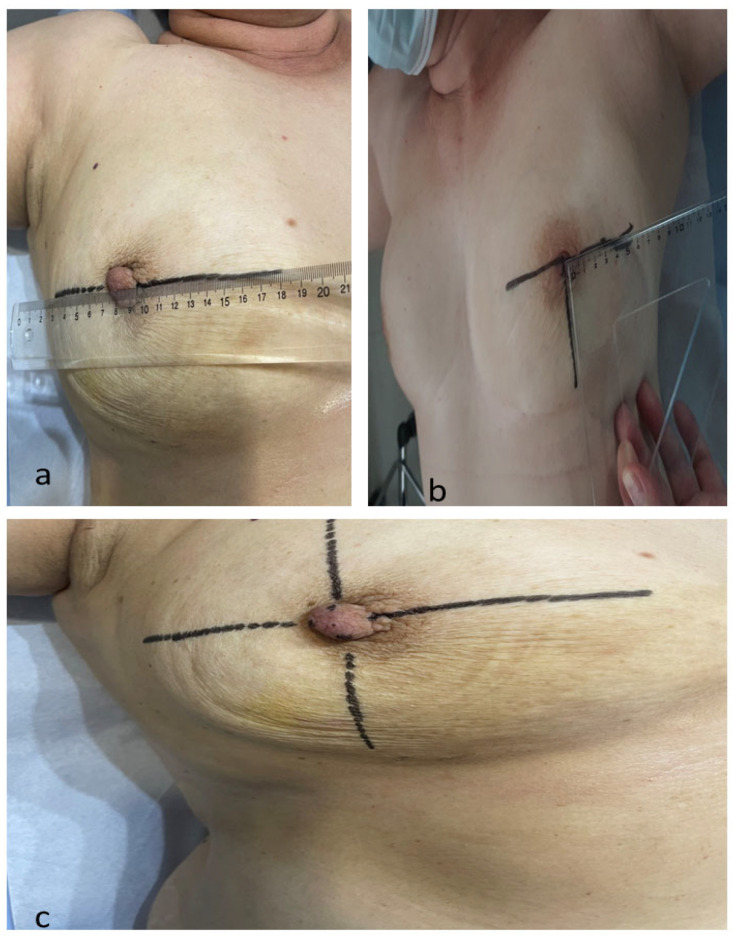
Drawing the horizontal and vertical axes on the skin of the breast. (**a**) The horizontal axis is drawn in a line connecting the nipples of both breasts. (**b**) The vertical axis is drawn at right angles to the horizontal axis. (**c**) A coordinate system of horizontal and vertical axes drawn on the skin of the breast, intersecting at the nipple.

Initially, a coordinate system of horizontal and vertical axes was drawn on the skin of the breast, intersecting at the nipple. The patient was positioned as if on an operating table, with her arms outstretched at right angles to the body axis (Figure 1a–c).

**Figure 2:**
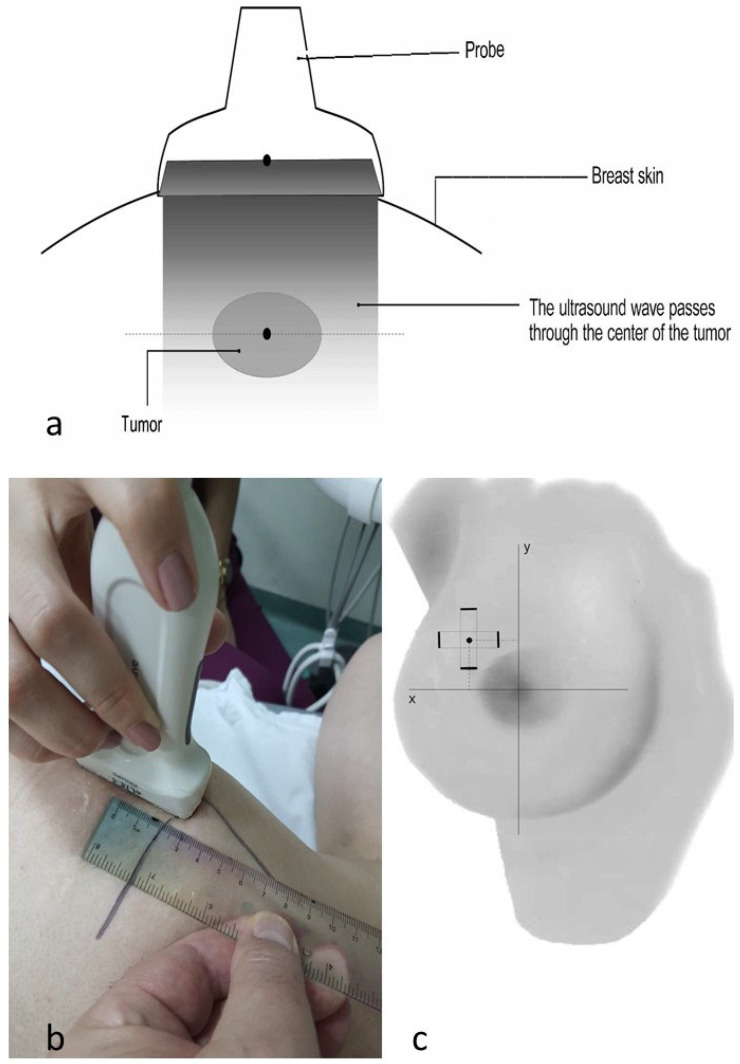
Determining of the projection of the central point of the tumor on the skin. (**a**) Ultrasound probe placed orthogonally to the surface of the skin above the tumor; the largest section of the tumor is in the center of the ultrasound screen. (**b**,**c**) The distances from the center of the ultrasound probe to the corresponding axes drawn on the skin, measured with a ruler; the long axis of the probe in both positions must be absolutely parallel with the “x” and “y” axes.

The central point of the tumor was determined by placing the ultrasound probe orthogonally to the surface of the skin above the tumor, ensuring that the largest section of the tumor was in the center of the ultrasound screen (Figure 2a).

The distance from the center of the ultrasound probe to the corresponding axis drawn on the skin was measured with a ruler and recorded. This procedure was performed in cranio-caudal and medio-lateral directions, requiring the probe to be absolutely parallel, first with the “x” and then with the “y” axis (Figure 2b,c).

The distance of the center of the probe from the “x” axis represented the coordinate of the tumor’s central point on the “y” axis (the numerical values for tumors localized in the upper quadrants were positive and those for tumors localized below the nipple were negative). The distance of the center of the probe from the “y” axis represented the coordinate of the tumor’s central point on the “x” axis (the numerical values for tumors localized to the right of the nipple were positive and those for tumors localized to the left of the nipple were negative).

**Figure 3:**
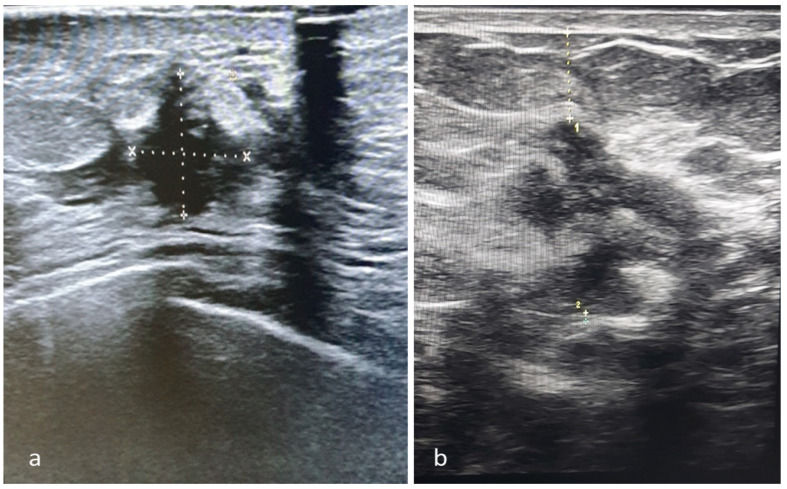
The ultrasound measurements. (**a**) Largest horizontal (craniocaudal and mediolateral) and vertical diameters of the tumor. (**b**) Distances of the superficial margin of the tumor–skin (1) and the deepest margin–pectoral fascia (2).

The ultrasound dimensions of the largest tumor diameters in the cranio-caudal and medio-lateral directions were determined during these measurements, alongside the vertical dimension (in the skin fascia direction), the distance from the tumor’s superficial margin to the skin, and the distance from the tumor’s deepest margin to the fascia, and these values were recorded (Figure 3a,b).

We obtained the following dimensions at the end of the procedure: the largest tumor diameters in the cranio-caudal and medio-lateral directions, the vertical tumor dimension, the distance from the tumor’s superficial margin to the skin, the distance from the tumor’s deepest margin to the fascia, and the coordinates of the tumor’s central point in relation to the coordinate system drawn on the breast skin (Table 1).

These numerical values fully defined the tumor’s three-dimensional position in the breast. The entire procedure was repeated every 6 weeks on average during NAST (after two cycles of AC, after four cycles of AC, and after six cycles of taxane) to observe the tumor regression pattern and potential spatial movements of the tumor caused by regression. Breast MRI was performed with each ultrasound positioning to verify the ultrasound assessments and to more reliably evaluate the tumor regression pattern—whether concentric or non-concentric (a fragmentation or honeycomb pattern) [19,20,30]. MRI use does not aim to position the tumor in the breast because of the different positions of the patient during the US and MRI examinations.

During positioning, the ultrasound probe must always be placed orthogonally to the skin surface and with the lightest possible pressure applied to the breast tissue to avoid spatial deformations due to bending the breast tissue.

### 3.2. Surgical Procedure

**Figure 4:**
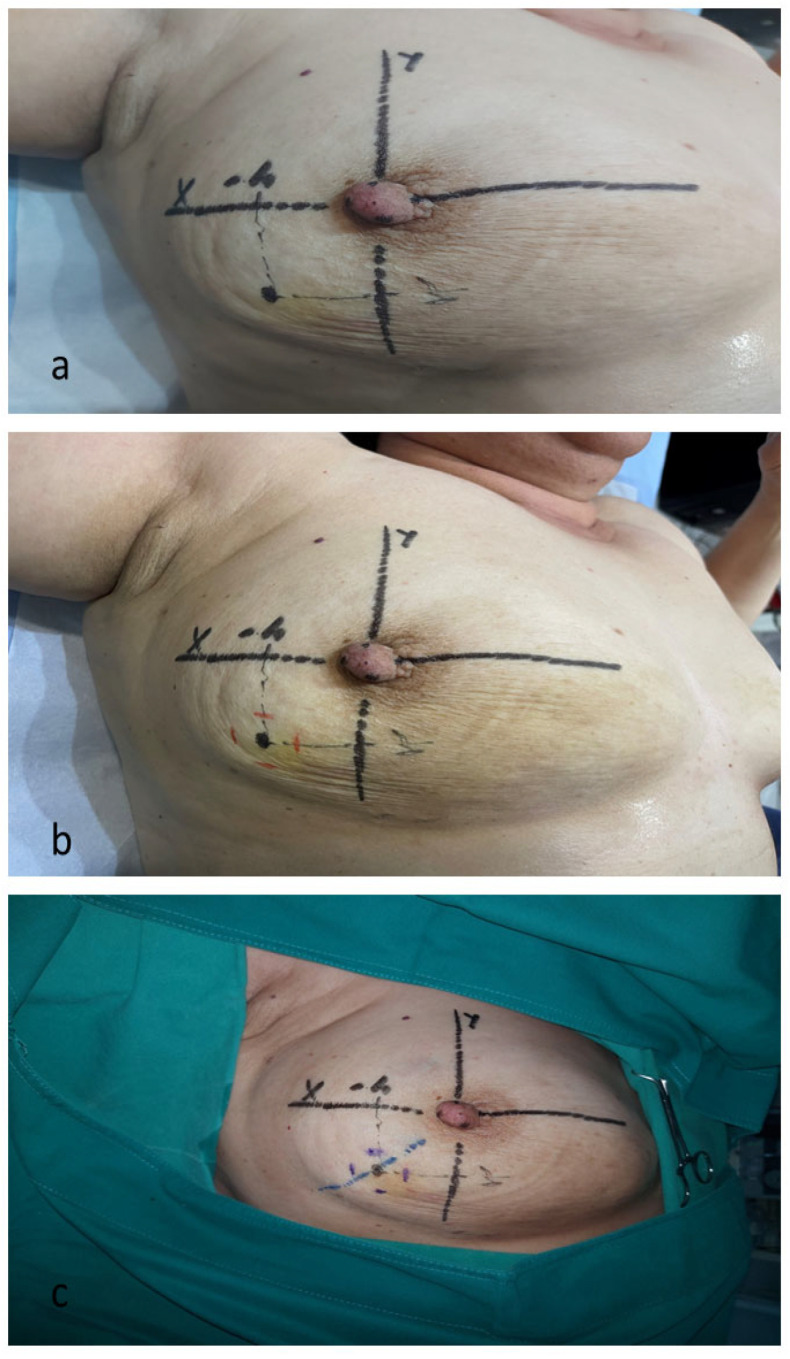
Planning of the surgical excision of the tumor bed based on the tumor positioning records. (**a**) Central point of the tumor drawn based on known coordinates. (**b**) Red dashes indicate tumor margins in the cranio-caudal and medio-lateral directions. (**c**) The blue dotted line indicates the skin incision line.

The procedure of drawing the coordinate system on the skin was repeated before performing surgery on the operating table. The projection of the pre-existing tumor’s central point was marked on the skin based on the previously determined coordinates. The tumor’s cranio-caudal and medio-lateral margins were marked on the skin based on the dimensions obtained during diagnostic positioning (Figure 4a,b).

The skin incision was planned given the tumor’s projection on the skin, which was obtained using the previously described measurements/techniques. A simple incision or excision of the part of the skin above the tumor was performed depending on the initial distance of the tumor’s superficial margin from the skin (if the distance of the tumor’s superficial margin from the skin was less than 10 mm) (Figure 4c).

**Figure 5:**
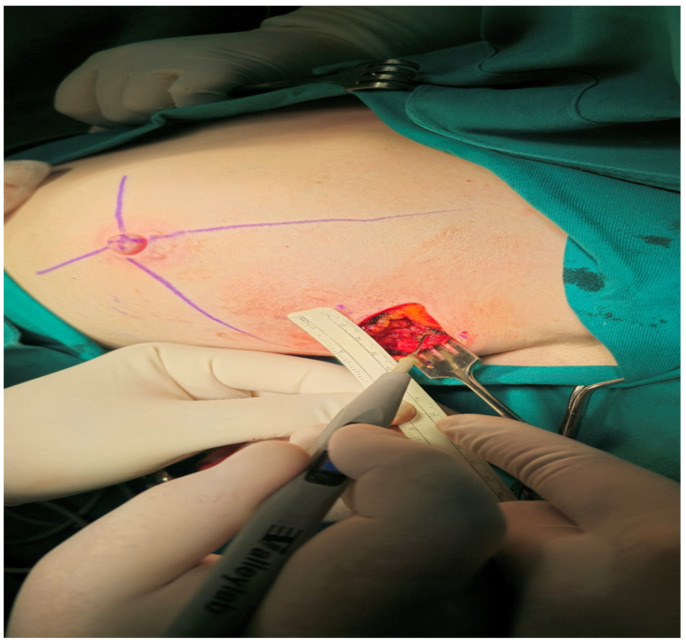
Measurement of spatial relationships by sterile ruler during the operation.

Surgical excision of the tumor bed was performed with maximal avoidance of excessive traction and tissue bending, with frequently repeated observations and measurements (by a sterile ruler) of spatial relationships during the operation. The vertical plan of the breast tissue resection followed the marked points on the skin representing the projections of the tumor edges in the cranio-caudal and medio-lateral directions (Figure 5).

The horizontal plan of the breast tissue resection (in the directions towards the skin and towards the pectoral fascia) was based on the diagnostically recorded distances of the superficial edge of the tumor from the skin and the deep edge of the tumor from the pectoral fascia. The resection margins during the operation were able to be additionally marked with ink, following projections of the diagnostically given coordinates. This procedure complements the visual orientation of the extent of the resection.

The extent of the resection depended on the tumor’s pre-NAST size and the tumor regression pattern during therapy. For tumors smaller than 3 cm in the largest diameter before NAST, the resection of the tumor bed was performed a few millimeters “outside” the projected margins of the initial tumor. For tumors with a largest diameter of more than 3 cm before NAST and with concentric regression established on MRI, the resection of the lodge followed the projected margins of the last tumor residue, as seen via ultrasound during NAST, in the same way as for tumors smaller than 3 cm. In cases where a honeycomb pattern of tumor regression was evaluated via MRI, the resection of the lodge followed the projected margins of the initial tumor, regardless of the tissue defect.

The specimen of the resected tissue was spatially oriented and delivered to the pathologist.

The excision of the tumor bed can be performed using oncoplastic surgery, or through a skin incision that is not placed directly above the tumor but eccentrically. The landmarks remain unchanged: the projection of the cranio-caudal and medio-lateral edges of the tumor on the skin and the distances of the superficial and deep edges of the tumor from the skin and pectoral fascia, respectively. The surgical technique is completely analogous to the excision of a palpable tumor, with the exception that previously determined measurements and coordinates are used as a resection guide instead of palpation. The bed excision of the tumor can be successfully performed by a standard-educated surgeon, consistently following the description of our previously presented technique. This indicates that the diagnostic procedure of tumor localization can be performed in one medical center and surgical excision of the tumor bed in the other.

## 4. Results

Ninety-three patients with 94 tumors (one patient had bilateral cancer) were recruited into this study. Fifteen patients (16%) were excluded due to inadequate ultrasound visualization of the tumor (11 pts.), constitutionally ptotic breasts unsuitable for our technique (2 pts.), or early tumor progression during NAST (2 pts.). Ultimately, 79 patients completed the entire procedure, in which 31 tumors (39%) with CCR after NAST were operated on using our tumor localization technique (Table 2).

Demographic and baseline disease characteristics of the patients are presented in Table 3.

A tumor size of less than 30 mm and positive Her2 receptor status were the most significant predictors of achieving CCR.

Breast-conserving surgery (BCS) was performed in all 31 operations, fulfilling the entire methodology of localization, excision, and specimen orientation. For two patients, the operation was later continued with mastectomies due to their wish to avoid irradiation, but this did not influence the study’s requirements for histopathological processing. In 24 of 31 operations, pCR was histologically verified, with the presence of histological signs of tumor regression in the excised specimens. Microscopic foci of residual tumor cells in the excised specimen were observed in seven patients, with a negative margin status in all seven cases.

Fragmented tumor regression (a honeycomb pattern), assessed via repeated MRI examinations, was verified in 3 out of 7 patients with microscopic residues and 7 out of 24 patients with pCR, totaling 10 out of 31 (32%).

The ratio of the resected specimen volume to the pre-NAST tumor volume was on average 0.91 (ranging from 0.1 to 2.38). Values of less than one were achieved for tumors in which excision of the tumor bed was performed based on the volume of the last residue of the tumor in regression visible via ultrasound during NAST (13 cases) (Table 4).

## 5. Discussion

The tumor’s location in the breast before NAST must be specified because a significant proportion of well-responsive tumors become clinically (in palpatory and radiological ways) undetectable after NAST. The lack of a precise orientation regarding the pre-existing tumor’ location in the breast prevents the surgeon from performing an oncologically adequate breast-conserving excision of the tumor site, often leading them to perform a mastectomy [5].

Oncologically adequate surgery remains the conditio sine qua non of successful breast cancer treatment; therefore, it is more important than the esthetic effects of surgery. The occurrence of local–regional recurrence, most often a consequence of inadequate or insufficient surgery, reduces the fifteen-year survival rate by as much as 25% among patients with local recurrence [31]. Paradoxically, NAST has a negative effect on the local control of the disease. Post-NAST patients have a significantly higher percentage of local recurrences than patients who are primarily treated surgically at the same stage [32]. The reason for this “paradox” could be subjective: tumor reduction after NAST motivates surgeons to perform breast-sparing surgery to a significantly higher percentage than in patients primarily operated on at the same initial stage. However, this conservative surgery can generate an invalid perception of the regressed tumor zone. The macroscopic impression differs from the microscopic fact. CCR (tumors are undetectable via palpation and radiology) does not always correlate with pCR, and an achieved pCR does not guarantee the absence of local–regional recurrence or systemic disease progression [33]. These facts emphasize the importance of adequate surgical treatment of breast carcinoma after NAST [34].

The adequacy of surgery on a disappeared tumor site depends on the adequacy of localizing the tumor before its disappearance. Standard surgical techniques for BCS after CCR achieved via NAST rely on the pre-NAST localization of the tumor by invasive insertion of various markers into and around the tumor [6,8,9,10,11,12,13,14,15,16,17,18,19,20,21,22,23]. Applying these techniques requires trained staff and equipment for invasive marking, which is lacking in many medical centers dealing with breast cancer surgery [5]. One-time marking before starting NAST limits the tailoring of the surgery as per the dynamics and pattern of tumor regression during NAST [35]. Newer proposed non-invasive tumor localization techniques using MRI [36] could be insufficient due to the discrepancy (mismatch) in the breast position during MRI diagnosis and the surgical procedure. The disadvantages of invasive marking techniques mentioned in the Introduction have been described in studies dealing with the results of their applications [6,8,9,10,11,12,13,14,15,16,17,18,19,20,21,22,23]. The frequency of these disadvantages, the statistical significance of this frequency, their clinical implications, and other details are insignificant in terms of quantitatively comparing these disadvantages with our non-invasive three-dimensional tumor localization technique because it does not possess these disadvantages.

Our original technique of the millimeter-precise determination of the position of the tumor in the breast in three dimensions is based on ultrasound measurements in relation to known and constant (unchangeable) coordinates. It can be repeated multiple times during NAST. The patient and breast positions are the same during the measurements and the operation. Applying this technique does not require additional equipment and consumables for invasive marking. The detailed description of the technique that we have provided is sufficient for its performance by standard-educated radiologists and surgeons without needing additional education. The technique is non-invasive, poses no risk of complications such as bleeding and infection, and it is comfortable for the patient. Moreover, our technique is not competitive with other techniques; it can be applied alongside them. The initial results illustrate the technique’s feasibility; their purpose is not to draw definitive conclusions about its validity.

Clinically detectable residues of tumor tissue represent the most precise landmark for the place and extent of excision [33]. Marking the tumor position before and during NAST helps the accuracy of surgery only in the absence of these clinically detectable landmarks. Therefore, we tested the clinical benefit and precision of our localization technique only in patients with CCR, that is, in patients who did not have palpable and radiologically detectable manifestations of a pre-existing tumor. In our study, the percentage of utilization of localizations prior to and during NAST was 39% of all tumors that had partial or complete regression, meaning this proportion of tumors was clinically undetectable after NAST.

In a recent systematic review of pre-NAST tumor-marking techniques, the authors used margin status as the primary outcome measure in assessing the success of tumor marking [28]. Patients with pCR were even excluded from analyses of margin status, since “patients with pCR cannot have an unsatisfactory margin”. This is, in a sense, opposed to our concept, which starts from the fact that the usefulness of tumor marking increases proportionally if there is less residual tumor tissue in the tumor bed, because residual tumor tissue is a satisfactory marker in itself. In cases of achieved pCR, there is the lowest quantity of residues that would serve as a marker, so an alternative marker is most needed, and its usefulness is most clearly observed in such cases.

The verification of the clinical benefit of marking, i.e., the precision of surgery based on the localized tumor position prior to and during NAST, in our paper primarily relies on histological parameters that confirm that the lodge of the pre-existing tumor was successfully excised: histologically verified residual tumor cells or secondary histological indicators of a pre-existing tumor that has completely regressed. Margin status, defined as the presence of histologically unchanged breast tissue in all directions around the target focus, as well as the volume ratio of the excised specimen and the pre-NAST tumor, represent secondary parameters of surgical precision. Based on the initial results of the practical application of our technique, 100% efficiency was achieved in the precision of the excision of the pre-existing tumor bed and the achievement of negative margins, with a satisfactory ratio of the volume of the initial tumor to the volume of the excised specimen. The small number of patients and the lack of a control group are not limitations of our study, since the aim of our study was to test the feasibility of the technique and not to draw definitive conclusions about its validity. The definitive test of the quality of surgical excision will be local control of the disease, based on five-year follow-ups [9].

An earlier non-invasive skin tumor projection tattoo technique before NAST, proposed by Lannin et al. [7], is based on a one-time pre-NAST marking of the tumor’s projection on the skin of the breast as determined via palpation or ultrasound. This technique was compared with a metal-clip marking technique in Espinosa-Bravo et al.’s [8] study. The study showed that operations guided with the tattoo technique led to a significantly larger volume of excised tissue, adding no benefits to the surgical margins. Our original technique of defining the three-dimensional tumor position in the breast before and during NAST is also non-invasive. It has two advantages compared with the skin tattoo technique: (1) determining the tumor position in three dimensions and (2) the dynamic monitoring of possible displacements of the tumor position due to regression during NAST.

The successive monitoring of the regression pattern during NAST with MRI, which we recommend, contributes to the precision of the technique. The dynamic monitoring of tumor regression during NAST represents a general advantage over one-time marking before NAST, which includes all invasive marking techniques.

Our prospective study continues with the follow-up of operated patients and the inclusion of new patients, in whom the standard pre-NAST clip marking technique and our technique are simultaneously applied in order to directly compare the accuracy of invasive and non-invasive marking.

### 5.1. Limitations of the Presented Technique

The standard limitations of our technique are common to ultrasound-guided tumor-marking techniques and pertain to the difficulty of ultrasound visualization of tumors. Some tumors are difficult to differentiate from the surrounding breast tissue using ultrasound due to their structure, so determining of their edges is unreliable [37]. In our study, 11 of 94 tumors (11.7%) were excluded for this reason.

Specific difficulties in performing localization and subsequent surgical excision originate from the consistency of breast tissue, which bends and changes spatial relationships under the pressure of an ultrasound probe or during surgical work. This is especially pronounced in voluminous and extremely ptotic breasts. Only two patients (2%) were excluded for this reason. Inaccuracies of this origin can be minimized via extremely gentle manipulations during positioning and surgical excision, as emphasized in the technique description.

Two patients (2%) were excluded due to disease progression under NAST, which cannot be included with our technique’s limitations.

This paper did not cover the spatial orientation and positioning of the positive axillary lymph nodes. However, this technique can be applied to axillary lymph nodes by precisely recording the place and angle at which the ultrasound probe lies on the skin while visualizing a positive lymph node.

Frequent MRI examinations somewhat complicate the procedure and increase its cost; they are unnecessary but contribute to the technique’s precision and reliability.

Another limitation is the greater number of diagnostic procedures during NAST compared to the standard regimen, causing additional burden on radiology departments and patient discomfort. This limitation is conditional; the proven clinical utility for the patient in terms of disease control exceeds this type of limitation.

### 5.2. Advantages of the Presented Technique

The advantages of our technique over standard tumor-marking procedures stem from overcoming and neutralizing the shortcomings of standard pre-NAST tumor-marking techniques. The general advantages of our technique include the ability to perform multiple repetitions during NAST and its lack of competitiveness with standard techniques, whereby it can be used as a supplement to standard invasive marking techniques. They include the following:

- The incorporation of the third dimension and objective multiple ultrasound measurements of tumor localization during NAST. Lannin et al. [7] performed excisions based on a one-time pre-NAST projection of the tumor on the breast skin, which potentially unnecessarily increases the volume of the excised tissue. The percentage of positive resection margins in that study was 10%, while our study featured no positive resection margins.
- The avoidance of complications associated with invasive marking procedures: hematoma, infection, marker misplacement, marker migration, and poor marker visibility at the time of surgery. Every study addressing the problem of invasive tumor marking states the presence of a certain percentage of these complications, which threaten the precision of surgery [26] or require preoperative WNL [27]. Our technique does not encounter complications of this type.
- Simplicity and cost–benefit ratio. Our technique can be implemented by a radiologist or surgeon who is not trained in invasive marking techniques, and it can be applied in health centers lacking specific marking equipment, avoiding additional financial outlays and inconveniences associated with invasive marking.

## 6. Conclusions

We presented a detailed description of an original technique for the non-invasive three-dimensional localization of tumors in the breast before starting neoadjuvant systemic therapy, aiming to optimize surgery after the tumor’s complete clinical regression.

Based on the initial results, the described technique is feasible and demonstrates a satisfactory level of oncological reliability and esthetic surgical outcomes.

The potential advantages over standard marking techniques are based on non-invasiveness, simplicity, and the possibility of multiple repetitions during neoadjuvant systemic therapy.

Our prospective study continues with the inclusion of new patients, in whom the standard pre-NAST clip marking technique and our technique are simultaneously applied to compare their accuracy.

## Figures and Tables

**Table 1:** Standard record of performed tumor positioning—example of a tumor located in the lower inner quadrant of the right breast.

Tumor Positioning Record		
Name and Surname:		
Status of Neoadjuvant therapy:	Before starting therapy	
	After ________________ cycles of NAHT	
Breast:	Right	Left ○
Date of examination:		
Tumor dimensions:	1. cranio-caudal:	22 mm
	2. medio-lateral:	18 mm
	3. vertical:	28 mm
Distance between superficial margin of the tumor and the skin:	6 mm	
Distance between deepest margin of the tumor and pectoral fascia:	0 mm	
Coordinates of tumor’s central point on the skin:	X axis	+32 mm
	Y axis	−22 mm

**Table 2:** Numerical framework of the study.

Description	Number of Patients (%)
Total patients included	93 (94 tumors) (100%)
Patients on whom the entire procedure was completed	79 (84%)
Excluded due to unsuitability for the procedure	15 (16%)
Patients with CCR * who underwent surgery using our technique	31/79 (39% of those where the procedure was completed)

* CCR—complete clinical response.

**Table 3:** Demographic and baseline disease characteristics of the patients.

Characteristic	Patients in the Study (*N* = 79)	
Response to NAST—no. of patients (%)	CCR achieved 31 (39)	No CCR 48 (61)
Age—no. of patients (%)		
<50 yrs	16 (20)	21 (27)
>50 yrs	15 (19)	27 (34)
US tumor size—no. of tumors (%)		
18–30 mm	22 (28)	8 (10)
31–47 mm	9 (11.4)	40 (50.6)
Hormone receptor status—no. of patients (%)		
Positive	16 (20)	30 (38)
Negative	15 (19)	18 (23)
Her2 receptor status—no. of patients (%)		
Positive	14 (18)	13 (16.5)
Negative	17 (21.5)	35 (44)
Nodal status—no. of patients (%)		
N0	13 (16.5)	20 (25)
N1	18 (23)	28 (35.5)
Triple negative—no. of patients (%)	5 (6)	6 (7.6)

**Table 4:** Surgical and histological parameters of the performed procedure.

Parameter	Mastectomy	BCS (Breast-Conserving Surgery)
Number (%)	2 (6.5%)	29 (93.5%)
pCR (Pathological Complete Response)	2	22
Residual Microfoci of Carcinoma	0	7
Positive Margin	0	0
Histological Signs of Pre-existing Tumor	2 (100%)	29 (100%)
Concentric Regression Pattern	2	19
Non-Concentric Regression Pattern	0	pCR 7/22, non-pCR 3/7
Ratio of Excised Specimen Volume to Pre-NAST Tumor Volume	X	0.91 (0.01–2.38)

## Data Availability

The data that support the findings of this study are available from the corresponding author upon reasonable request.

## Abbreviations

NAST	Neoadjuvant systemic therapy
pCR	Complete pathohistological response
CCR	Complete clinical regression
BCS	Breast-conservative surgery
ROLL	Radioguided occult lesion localization
WNL	Wire-needle localization
